# Novel Aspects of Extracellular Vesicles in the Regulation of Renal Physiological and Pathophysiological Processes

**DOI:** 10.3389/fcell.2020.00244

**Published:** 2020-04-15

**Authors:** Juan Pablo Rigalli, Eric Raul Barros, Vera Sommers, René J. M. Bindels, Joost G. J. Hoenderop

**Affiliations:** ^1^Department of Physiology, Radboud Institute for Molecular Life Sciences, Radboud University Medical Center, Nijmegen, Netherlands; ^2^Department of Endocrinology, School of Medicine, Pontificia Universidad Católica de Chile, Santiago, Chile

**Keywords:** extracellular vesicles, exosomes, renal physiology, renal disorders, renal injury

## Abstract

Extracellular vesicles (EV) are nanosized particles released by a large variety of cells. They carry molecules such as proteins, RNA and lipids. While urinary EVs have been longer studied as a source of biomarkers for renal and non-renal disorders, research on EVs as regulatory players of renal physiological and pathological processes has experienced an outbreak recently in the past decade. In general, the microenvironment and (patho)physiological state of the donor cells affect the cargo of the EVs released, which then determines the effect of these EVs once they reach a target cell. For instance, EVs released by renal epithelial cells modulate the expression and function of water and solute transporting proteins in other cells. Also, EVs have been demonstrated to regulate renal organogenesis and blood flow. Furthermore, a dual role of EVs promoting, but also counteracting, disease has also been reported. EVs released by renal tubular cells can reach fibroblasts, monocytes, macrophages, T cells and natural killer cells, thus influencing the pathogenesis and progression of renal disorders like acute kidney injury and fibrosis, nephrolithiasis, renal transplant rejection and renal cancer, among others. On the contrary, EVs may also exert a cytoprotective role upon renal damage and promote recovery of renal function. In the current review, a systematic summary of the key studies from the past 5 years addressing the role of EVs in the modulation of renal physiological and pathophysiological processes is provided, highlighting open questions and discussing the potential of future research.

## Introduction

Extracellular vesicles (EVs) are a group of membrane-enclosed nanosized particles released from a large variety of cell types (Théry et al., 2019). EVs are classified according to their origin as exosomes for those of endosomal origin and microvesicles for plasma membrane-derived vesicles. All vesicles incorporate molecules from their cell of origin, including proteins (Pisitkun et al., 2004), nucleic acids (Miranda et al., 2010) and lipids (Del Boccio et al., 2012). In the kidney, research has focused mainly on urinary EVs (uEVs), released by the tubular and urinary tract cells, and their potential as biomarkers of kidney-related diseases (Street et al., 2017). However, in the last 5 years more interest has been placed in the role of EVs as essential regulators in renal physiology and pathophysiology (Wang et al., 2017). In this review, the latest discoveries on the regulatory role of EVs in health and disease are discussed.

For the sake of clarity, and in agreement with the International Society for EVs (Théry et al., 2019), the term EV for the different populations of vesicles will be used, since isolation methods and markers do not allow to fully distinguish exosomes from other vesicles.

## Evs in the Regulation of Physiological Processes in the Kidney

### EV-Mediated Intrarenal Communication

EVs released by different segments of the nephron carry RNAs and proteins capable of communicating signals in an upstream to downstream fashion within the nephron Alvarez et al., (2012). For instance, (Gildea et al., 2014) described the uptake of proximal tubule EVs by distal convoluted tubule and collecting duct cells as well as by the same proximal cells. Furthermore, it was reported that decrease in reactive oxygen species (ROS) production in proximal tubule cells by dopaminergic stimulation was transferred to distal tubule- and collecting duct cells by EVs secreted by the proximal cells. Conversely, the increase in ROS in proximal tubule cells exposed to angiotensin II was not transferred via EVs to cells downstream (Gildea et al., 2014). This observation indicates certain specificity of EV-mediated communication toward particular treatments and phenotypes. From the cellular point of view, the cargo of the EVs might be specifically influenced by particular stimuli applied to the donor cell and therefore determine EV activity upon reaching the target cell.

**FIGURE 1:**
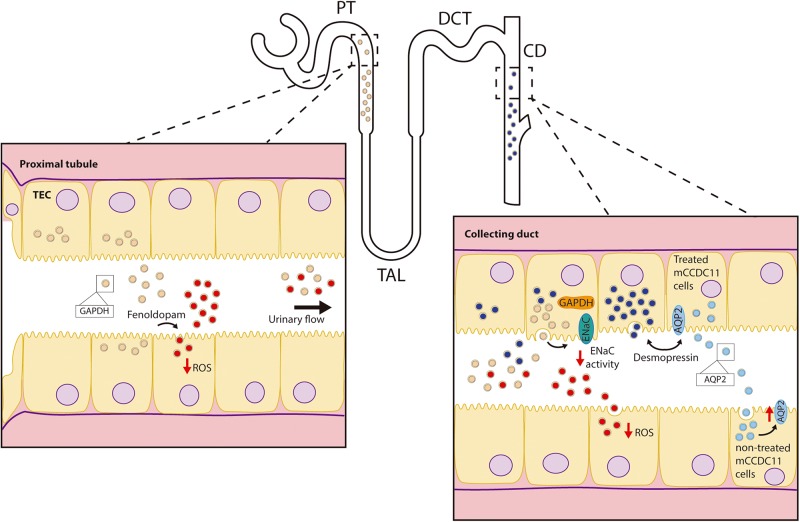
Role of EVs in renal physiology. Depicted are renal physiological processes mediated by EVs: down-regulation of ENaC activity in collecting duct cells by proximal tubule EVs; EV-mediated transfer of the decrease in intracellular ROS by fenoldopam in the proximal tubule to the collecting duct, and the stimulation of EV uptake as well as the release of EVs with higher levels of AQP2 by collecting duct cells exposed to desmopressin. AQP2-enriched EVs increased water permeability of other (target) collecting duct cells.

A more recent study demonstrated that treatment of cortical collecting duct cells with desmopressin (i.e., synthetic vasopressin analog) stimulated uptake of EVs released by proximal tubule and collecting duct cells (Oosthuyzen et al., 2016). An overview on renal physiological processes mediated by EVs and the bioactive components within the EV cargo is graphically depicted in Figure 1.

### Regulation of Tubular Transport Processes

Within the cargo of renal EVs, different ion-, organic compound- and water transporters, their codifying RNAs, as well as microRNAs (miRNAs) targeting their expression can be found. In this regard, EVs from cortical collecting duct cells carry higher protein levels of aquaporin 2 (AQP2) after exposure of the donor cells to desmopressin (Street et al., 2011). Moreover, these EVs had the ability to transfer functional AQP2 to other collecting duct cells. Since AQP2 plays a key role in water reabsorption in this segment and, thus, in the concentration of urine (van Lieburg et al., 1995), increased water permeability due to exposure to EVs could be expected. These observations suggest a potential role of EVs in the mediation of physiological responses after changes in the hydration status (Street et al., 2011).

Additionally, a comprehensive study using next-generation sequencing evaluated the diversity of miRNAs present in uEVs and their impact on the expression of renal transporters and channels (Gracia et al., 2017). Based on the 10 most abundant miRNAs found (miR-10b-5p, miR-10a-5p, miR-30a-5p, miR-26a-5p, miR-22-3p, miR-204-5p, miR-181a-5p, miR-27b-3p, miR-30d-5p, miR-192-5p), the renal outer medullary potassium channel (ROMK, Kir1.1), the plasma membrane calcium-transporting ATPase (PMCA1) and the amino acid transporter SNAT2 (*SLC38A2*) appeared as candidates to be regulated by these EVs. In fact, addition of the vesicles to the human proximal cells HKC-8 resulted in a decrease in the mRNA and protein levels of SNAT2. The decrease in *SLC38A2* mRNA levels suggests lower mRNA stability due to the presence of targeting miRNAs in the vesicles. Similarly, PMCA1 and ROMK protein expression were down-regulated by uEVs in human collecting duct (HCD) cells (Gracia et al., 2017). This report indicates a potential regulatory role of EVs also in calcium and potassium reabsorption. Additionally, the transport of amino acids may be regulated by EVs.

The epithelial sodium channel (ENaC) is expressed in the distal part of the nephron and plays a significant role in sodium homeostasis. Jella et al., (2016) described an acute inhibition of ENaC activity in collecting duct cells after exposure to EVs released from proximal cells. The effect was observed majorly for apical vesicles, thus indicating a potential proximal to distal communication mechanism along the nephron via pro-urine flow. The authors attributed the inhibitory action to EV-carried glyceraldehyde-3-phosphate-dehydrogenase (GAPDH), as immunoprecipitation studies demonstrated the physical interaction between GAPDH and ENaC.

### Regulation of Renal Blood Flow

A recent study showed in a mouse model that application of acupuncture with low frequency electrical stimulation (Acu/LFES) to the hindlimb muscles increases renal blood flow, compared to mice treated with acupuncture without electrical stimulation (Su et al., 2018). Administration of the inhibitor of exosome release GW4869 (Menck et al., 2017) prevented the increase in the blood flow by Acu/LFES. Further mechanistic information was obtained using miRNA deep sequencing analysis, which displayed increased levels of miR-181d in serum EVs from Acu/LFES mice. Subsequently, binding of miR-181d to the 3’UTR of angiotensinogen mRNA and lower angiotensinogen levels were observed for Acu/LFES, probably accounting for the hemodynamic effects described above (Su et al., 2018). These findings point EVs as an additional factor regulating renal blood flow. Moreover, the described study provides a proof-of-concept for EV-mediated communication at a systemic level with the kidney as a target.

### Organogenesis

Nephrogenesis requires a complex exchange from inductive signals between the ureteric bud (UB) and the metanephric mesenchyme (MM) in which the activation of the Wnt pathway in the latter plays a vital role (Wang et al., 2018). Hereby, a stimulatory effect of UB-derived EVs on the formation of pre-tubular aggregates in MM organoids has been described. Mechanistically, MM cells take up UB-derived EVs carrying miR-27a/b, miR-135a/b, miR-155, and miR-499. These miRNAs target the complex of APC (adenomatous polyposis coli), axin, GSK3β (glycogen synthase kinase 3), and CK1α (casein kinase 1α) and, thus, stimulate the nuclear accumulation of β-catenin (Krause et al., 2018).

## Evs in the Regulation of Renal Pathophysiological Processes

### Kidney Injury and Regeneration

**FIGURE 2:**
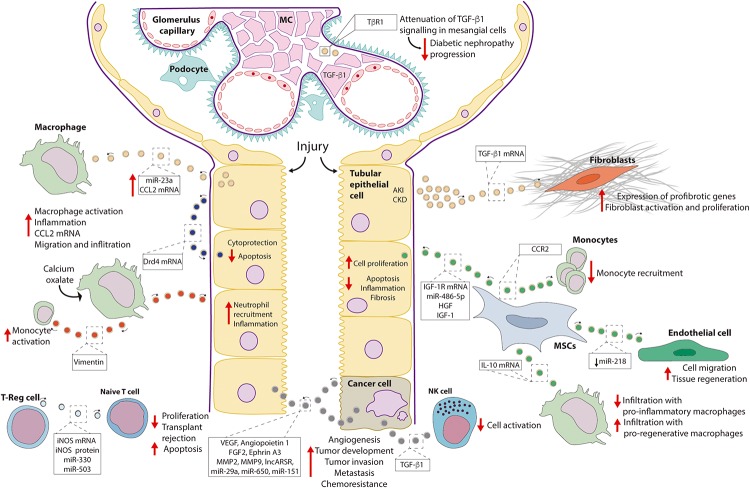
Role of EVs in renal pathophysiology. Depicted are renal pathophysiological processes mediated by EVs and, if known, the component of the EV cargo responsible for the effect. Abbreviations: CCL2, chemokine ligand 2; CCR2, chemokine receptor type 2; Drd4, dopamine receptor D4; FGF2, fibroblast growth factor 2; HGF, hepatocyte growth factor; IGF-1, insulin-like growth factor 1; IGF-1R, insulin-like growth factor 1-receptor; iNOS, inducible nitric oxide synthase; lncARSR, long non-coding ARN activated in in renal cell carcinoma with sunitinib resistance; MC, mesangial cells; MMP, matrix metalloproteinase; MSC, mesenchymal stem cells; NK, natural killer cells; TGF-β1, transforming growth factor β1; TβR1, TGFβ-receptor 1; T-reg, T-regulatory cells; VEGF, vascular endothelial growth factor.

Acute kidney injury (AKI) is characterized by the coexistence of damage and counteracting regenerative processes. So far, there is abundant evidence supporting the participation of EVs, both stimulating the progression of the injury as well as playing a cytoprotective role and promoting tissue regeneration. In this regard, the different cargo content of the vesicles could be the key to explain these opposing effects. The latest findings on the participation of EVs in renal injury are discussed here. The reviewed data are depicted in Figure 2.

#### Role of EVs Promoting Renal Injury

Tubulointerstitial inflammation is a complication of AKI. Mice models of ischemia-reperfusion injury and unilateral ureteral obstruction exhibited a higher content of miR-23a in EVs released by tubular cells compared to sham-operated animals. In particular, miR-23a downregulated the protein A20, a negative regulator of NFκB signaling, finally leading to macrophage activation (Li et al., 2019). In addition, an increase in chemokine ligand 2 (CCL2) mRNA content was observed in EVs from kidneys of mice exposed to lipopolysaccharide and in uEVs of rats with 5/6 nephrectomy, experimental models of AKI and chronic kidney disease (CKD), respectively (Lv et al., 2018). Hereby, filtered albumin is likely to be a factor directing changes in the EV cargo, as exposure of tubular epithelial cells (TECs) to albumin *in vitro* reproduced the increase in *CCL2* mRNA content observed in the vesicles *in vivo*. Furthermore, macrophage migration was more stimulated by EVs from albumin-treated TECs than by control EVs.

Fibroblast proliferation and activation are associated with renal fibrosis. A study with a mice model of AKI (unilateral ureteral obstruction) described higher levels of TGF-β1 (transforming growth factor β1) mRNA in renal EVs respect to control animals. Addition of these EVs to fibroblasts led to higher proliferation and up-regulation of α-smooth muscle actin (α-SMA) (Borges et al., 2013). In addition, EVs from mouse and human TECs exposed to hypoxic conditions also exhibited a higher potential to induce fibroblast proliferation and activation. These effects were prevented by transfection of donor cells with a siRNA against TGF-β1 (Borges et al., 2013).

EVs may also inhibit renal regeneration. The epidermal growth factor (EGF), which signals through the epidermal growth factor receptor (EGFR) is an essential molecule for epithelial regeneration. A study using a mouse proximal tubule cell line described stimulation of EV release by scratch wounding. Treatment with agonists and inhibitors of the EGF signaling pathway supported an inverse association between EV release and EGFR-mediated wound healing. In line with this, inhibition of exosome release with GW4869 and manumycin A increased EGFR activation and wound healing. These findings provided novel evidence that EVs may not only instigate the injury process but may also inhibit physiological regeneration mechanisms (Zhou et al., 2017).

#### Cytoprotective Effect of EVs

TGF-β1 signaling is necessary for extracellular matrix secretion by mesangial cells, and thus, for glomerulosclerosis and diabetic nephropathy progression. In this regard, clearance of the TβR1 receptor (i.e., TGF-β1 receptor) by loading into EVs has been demonstrated as a mechanism mediating down-regulation of TGF-β1 signaling in mesangial cells (Van Krieken et al., 2017).

EVs released by mesenchymal stem cells (MSCs) can contribute to tissue regeneration. For instance, EVs from human bone marrow MSCs increased proliferation of proximal tubule cells exposed to the nephrotoxic agent cisplatin. Beneficial effects of the EVs were prevented by silencing IGF-1R (insulin-like growth factor 1-receptor) in the donor cells (Tomasoni et al., 2013). A further study suggested a beneficial role of EVs from bone marrow MSCs by buffering extracellular levels of free chemokine ligand 2 (CCL2), thus preventing monocyte activation. The effect was attributed to the chemokine receptor type 2 (CCR2) present in the EVs (Shen et al., 2016). Likewise, improved renal function and reduced macrophage infiltration was observed in a mice model of diabetic nephropathy administered intravenously with EVs from bone marrow MSC. Beneficial effects may be explained by the down-regulation of adhesion molecules for macrophages (e.g., ICAM1) in the endothelial cells of the glomeruli and the peritubular capillaries by the EVs (Nagaishi et al., 2016).

Similarly, in a study using a model renal artery stenosis in pigs with metabolic syndrome, treatment with adipose tissue MSC EVs improved renal function. Reduced renal infiltration by pro-inflammatory macrophages and increased infiltration by pro-regenerative macrophages was observed. Effects were dependent on the presence of the anti-inflammatory cytokine interleukin-10 (IL-10) mRNA in the EVs (Eirin et al., 2017).

The endothelium of blood vessels constitutes a third source of MSC EVs. An analysis of miRNAs enriched in renal artery-derived vascular progenitor cells (RAPCs) highlighted the miR-218, which targets Robo1, a protein linked to endothelial cell migration. Interestingly, while under control conditions, RAPC release EVs enriched in miR-218, suppressing Robo1 expression and cell migration, exposure of RAPC to pro-oxidant conditions (i.e., tissue damage) results in a decrease in the content of miR-218 in the vesicles and, therefore, Robo1 up-regulation and increase of endothelial cell migration (i.e., regeneration of blood vessels) (Pang et al., 2017). In line with these observations, another related study reported the potential of EVs from human cord endothelial colony forming cells (ECFCs) to attenuate renal damage in a mouse model of ischemia-reperfusion (Viñas et al., 2016). Here, miR-486-5p was demonstrated as the critical component of the EVs improving renal function and reducing inflammation (Viñas et al., 2016).

### Nephrolithiasis

Recently, some lines of evidence pointed to the role of EVs in the pathogenesis of nephrolithiasis. For instance, a study evaluating the effect of different concentrations of calcium oxalate, one of the main components of kidney stones, on proximal cells, reported an increase in the release of EVs by elevated levels of oxalate (He et al., 2017).

Interstitial deposition of calcium oxalate constitutes one of the complications of nephrolithiasis. Hereby, macrophages seem to play a dual role in removing deposited crystals but also promoting inflammation. EVs isolated from macrophages previously exposed to calcium oxalate exhibited a different cargo of proteins with respect to EVs from control macrophages (Singhto and Thongboonkerd, 2018). When TECs were exposed to EVs from calcium oxalate-treated macrophages, increased inflammation and neutrophil recruitment were observed (Singhto and Thongboonkerd, 2018). Furthermore, EVs from calcium oxalate treated cells exhibited a higher capacity to bind crystals promoting their dissemination into the extracellular matrix, ultimately contributing to interstitial deposits (Singhto and Thongboonkerd, 2018). Interestingly, most of the proinflammatory effects were abolished by knock-down of vimentin in the donor macrophages, clearly indicating a role of this protein in the observed effects (Singhto et al., 2018).

### Renal Transplant Rejection and Tolerance

Organ rejection represents a common complication of transplanted patients. Participation of EVs stimulating allograft tolerance has been proposed. Specifically, EVs released by T regulatory (T-reg) cells have been reported to inhibit proliferation and stimulate apoptosis of naïve T cells *in vitro*. Inducible nitric oxide synthase (iNOS) mRNA and protein, which were enriched in the EVs, as well as miR-330 and miR-503 were necessary mediators of these effects. Noteworthy, a similar effect was also observed in a rat model of kidney transplantation, where intravenous and intrasplenic administration of EVs from T-regs improved renal function and prolonged allograft survival (Aiello et al., 2017).

Conversely, a role of EVs promoting host immune response and rejection has also been described. Patients diagnosed with transplant glomerulopathy after receiving a kidney transplant had circulating EVs with increased expression of fibronectin and collagen-IV (Sharma et al., 2018) and higher concentrations of antibodies against collagen IV and fibronectin (Angaswamy et al., 2014) compared with patients receiving a kidney transplant without this diagnosis. In this regard, a previous study with a rat model of renal transplantation showed that the immune response to collagen IV plays a role in allograft rejection (Joosten et al., 2002). Other studies pointed out a role of EVs released by endothelial cells in promoting inflammation and stimulating the immune response of the host (Cardinal et al., 2018). These findings not only suggest a potential role of circulating EVs in the prediction of transplant rejection but also raise possible therapeutic targets in order to counteract the rejection process.

### Renal Cancer

EVs can mediate intercellular communication also between tumor cells. In this regard, a pro-angiogenic and pro-metastatic role of EVs from CD105-positive cancer stem cells *in vitro* and *in vivo* has been described. Indeed, EVs derived from CD105-positive cells carried multiple proangiogenic mRNAs including VEGF, angiopoietin 1, fibroblast growth factor 2, ephrin A3, matrix metalloprotease 2, and matrix metallopeptidase 9. These factors were absent in EVs from CD105-negative cells. The same study also reported the presence of miRNAs with participation in tumor invasion and metastasis such as miR-29a, miR-650, and miR-151 in CD105^+^ derived EVs (Grange et al., 2011).

EVs released by renal cancer cells may also facilitate the evasion of the tumor cells from the immune system of the patient. In this regard, an inhibitory action of EVs released by cells from clear cell renal cell carcinoma on natural killer cells was described. The effect was attributed to TGFβ1 carried by the EVs (Xia et al., 2017).

Furthermore, EVs play a key role in drug resistance in renal cancer. In this case, a long non-coding RNA, named lncARSR (i.e., lncRNA activated in renal cell carcinoma with sunitinib resistance) was described as the bioactive component of EVs mediating chemoresistance. Once in the target (sunitinib sensitive) cell, lncARSR competes with the action of miR-34a and miR-449 preventing down-regulation of the membrane proteins AXL and c-Met which, ultimately, mediate resistance to sunitinib (Qu et al., 2016).

## Discussion

Besides their well-acknowledged role as a source of biomarkers, the past decade has witnessed EVs emerge as key players in cell-cell communication at autocrine, paracrine, and systemic level. Although a vast volume of information is already available for non-renal tissues, their participation in renal pathophysiology is a relatively novel field. Also, the heterogeneous nature of the nephron epithelium has reduced the study of EV-mediated processes to the utilization of *in vitro* models or pooled samples (e.g., uEVs) where relevant regulatory phenomena may remain undiscovered. Hereby, the use of microperfusion techniques to isolate EVs from specific segments of the nephron may constitute a promising tool (Cheng et al., 2013). Furthermore, a vast amount of the experimental evidence available results from *in vitro* studies and, therefore, need validation *in vivo* or in more complex models in order to be translated into a clinical setting. In this regard, *organ-on-a-chip* platforms may allow to culture, and thus scrutinize the effects of EVs, released by a donor cell on a target cell also mimicking physiological flow conditions and combining different segments from the nephron in one platform.

Tackling EV participation orchestrating pathophysiological responses, for instance, in acute kidney damage, can constitute a therapeutic alternative to mitigate inflammatory damage and fibrosis. Moreover, enrichment of EVs in particular components may allow to target physiological processes with therapeutic purposes, stimulate EV-mediated regenerative mechanisms and counteract those situations where EV contribute to disease development. For instance, several studies highlighted a clear regenerative role of EVs obtained from MSC. In this regard, EVs isolated from bone marrow MSCs could be used as preventive and regenerative therapy in cases where acute kidney damage is expected (e.g., treatments with highly nephrotoxic drugs).

Research on the role of EVs in kidney function may lead to the identification of novel biomarkers. Although, uEVs constitute a well-acknowledged source of information with diagnostic potential, several studies also described a role of plasma EVs conveying information about renal physiological and pathophysiological status (reviewed in Erdbrügger and Le, 2016). Importantly, different isolation and analysis methods, as well as the different nature of biological samples constitute a limitation toward obtaining a reliable picture of EV-mediated processes. The implementation of normalization procedures, for instance, by using EV pools from the general population (Witwer et al., 2013) or EV-mimetic particles (Lozano-Andrés et al., 2019) may provide more comparable results for different isolation techniques.

In conclusion, a significant volume of high-level studies has appeared during the past years, describing the participation of EVs regulating the normal function of the kidney as well as promoting and counteracting pathophysiological responses. While this may already bear therapeutic potential, considering the high diversity of the EV cargo, future studies will probably find EVs involved in increasing processes within the kidney and mediating communication between the kidney and other organs. This would likely result in more mechanisms that could be targeted in the aim of developing novel therapeutic strategies.

## Author Contributions

JR, EB, and JH conceived the manuscript. JR, EB, and VS drafted the manuscript. JR, EB, VS, RB and JH edited the manuscript. All authors approved the final version.

## Conflict of Interest

The authors declare that the research was conducted in the absence of any commercial or financial relationships that could be construed as a potential conflict of interest.
